# 

**Published:** 1879-09

**Authors:** 


### Table of Contents.

ORIGINAL COMMUNICATIONS.

Quinine Rash. By R. J. Nunn, M.D., Savannah, Ga..................... 321
A Case of Head Injury, with Spontaneous Trephination. By R. W. Westmoreland, M.D., Atlanta, Ga.................................322

REPORTS OF SOCIETIES.

Detroit Academy of Medicine.................................................. 336
Philadelphia County Medical Society........................................ 353

SELECTIONS

The Essential Physiology of Respiration................................ 324
Quarantine an Advertiser...................................................... 332
The Communicable Fevers and their Treatment.......................... 342
Consumption—Drinks, Food, Bathing, Exerci e, Clothing and Treatment................................................................... 344
Molar Pregnancy—An Anomalous Case.................................... 349

EDITORIAL.

Professional Honorarium........................................................ 359
Hypodermic Inj ction of Morphia............................................ 360

BIBLIOGRAPHICAL.

Guide to the Examination of Urine........................................ 361
Physiology and Histology of the Cerebral Convolutions............ 361
Materia Medica and Therapeutics........................................... 362

EXCERPTA.

Lumbago................................................................................ 362
Treatment of Obstinate Vomiting by Small Doses of Iodide of Potassium................................................................. 364
The Neglect and the Value of Blistering................................ 365
Injections of Linseed Oil for the Cure of Chronic Cystitis........ 366
Croton Oil in Vævus............................................................. 366
Sclerotinic Acid in Hæmoptysis An Effective Cathartic............. 367
Pilocarpine in the Pruritus of Pregnancy................................. 368
The Treatment of Indolent Ulcers by Means of Sheet Lead......... 368
A Case of Long-continued Priapism Accompanying Leukæmia...... 369
Hydrate of Chloral and Bromide of Potash Enemata in the Vomiting of Pregnancy............................................................ 370
The Therapeutic Value of Croton Chloral ................................ 371
An Elephant's Gratitude to a Druggist.................................... 372
Ginger Beer. Turpentine as an External Application for Small-pox. 373
How to Preserve Cider........................................................... 374
Anti-Toothache .................................................................... 375
Meteorological Report........................................................... 377
Urinary Calculi. Red Cinchona............................................... 378
Action of Iodoform................................................................ 379
A New Antiseptic ................................................................. 380
The Classification of Medicines............................................... 381
Salicylic Acid in the Treatment of Lupus.................................. 382
Use of Pilocarpinum Muriaticum in Children's Diseases............. 383
Compound Tincture of Iodoform. Cough Mixture..................... 384

**MANUAL OF CHEMICO-PHYSICS.**

By J. H. LOGAN, A.M., M.D.

Now in press—will be ready at the opening of Atlanta Medical College. A small edition, only, published.

